# Matrix-assisted laser desorption/ionization mass spectrometry (MALDI) in Alzheimer’s disease: a scoping review of proteomic alterations in neurological tissues

**DOI:** 10.1590/1980-5764-DN-2025-0437

**Published:** 2026-07-24

**Authors:** Gustavo Hayasaki Vieira, Victor Herlys Gomes Braga, Isabela Maciel Mesquita, Samara Silva Rodrigues, Samira Dionísio Maia, Giovana de Oliveira Araújo, Isadora Marra de Sá Sousa, Amarildo Lemos Dias de Moura

**Affiliations:** 1 Pontifícia Universidade Católica de Goiás, Faculdade de Medicina, Escola de Ciências Médicas e da Vida, Goiânia GO, Brazil.; 2 Universidade Federal de Uberlândia, Faculdade de Medicina, Uberlândia MG, Brazil.

**Keywords:** Alzheimer Disease, Proteomics, Mass Spectrometry, Central Nervous System Diseases, Doença de Alzheimer, Proteômica, Espectrometria de Massas, Doenças do Sistema Nervoso Central

## Abstract

Matrix-assisted laser desorption/ionization mass spectrometry (MALDI-MS) has become a valuable tool for molecular mapping in neurological disorders. This scoping review synthesized current evidence on its use in proteomic analysis of neural tissues in Alzheimer’s disease (AD). From 1,419 screened records, 26 studies met the inclusion criteria. Findings highlight MALDI-MS’s capacity to detect beta-amyloid (Aβ) proteoforms, and tau protein post-translational modifications linked to AD. Its high spatial resolution enables region-specific molecular profiling, enhancing understanding of AD pathophysiology and supporting early biomarker discovery. The review underscores the translational potential of MALDI-MS in advancing targeted therapeutic development.

## RESUMO.

A espectrometria de massas por dessorção/ionização a laser assistida por matriz (MALDI-MS) tornou-se uma ferramenta valiosa para o mapeamento molecular em distúrbios neurológicos. Esta revisão de escopo sintetizou as evidências atuais sobre seu uso na análise proteômica de tecidos neurais na doença de Alzheimer (DA). De 1.419 registros triados, 26 estudos atenderam aos critérios de inclusão. Os achados destacam a capacidade da MALDI-MS para detectar protoformas de beta-amiloide (Aβ) e modificações pós-traducionais da proteína tau associadas à DA. Sua alta resolução espacial possibilita o perfil molecular específico por região, ampliando a compreensão da fisiopatologia da DA e apoiando a descoberta precoce de biomarcadores. A revisão ressalta o potencial translacional da MALDI-MS no avanço do desenvolvimento de terapias direcionadas.

## INTRODUCTION

Alzheimer’s disease (AD), the most prevalent form of dementia, causes cognitive decline that can impair daily functioning and accounts for most cases in individuals aged 65 and older^1^. Over the past decade, significant advances have been made in the development of biomarkers for the specific and early diagnosis of AD, including neuroimaging markers obtained through positron emission tomography (PET) scans with amyloid and tau tracers, as well as biomarkers found in cerebrospinal fluid and plasma^2^. In this context, imaging mass spectrometry (IMS) has been widely used to study the pathophysiology of AD in brain tissues, both in animal models and human samples^3^.

Matrix-assisted laser desorption/ionization mass spectrometry (MALDI-MS) has emerged as a powerful tool for the detection of molecular markers, playing an essential role not only in identifying the biomarkers associated with neurodegenerative diseases such as AD^4^ ^,^ ^5^ but also in exploring the molecular mechanisms that underlie disease progression. A particularly relevant aspect in this context involves post-translational modifications (PTMs), such as phosphorylation, ubiquitination, acetylation, and citrullination, which profoundly influence protein structure, function, and aggregation in neurodegenerative disorders. PTMs are key modulators of pathological processes, including tau hyperphosphorylation, amyloid-β aggregation, and neuroinflammatory responses, all of which are central to AD pathogenesis^6^ ^,^ ^7^ ^,^ ^8^ ^,^ ^9^. Recent proteomic studies using MALDI-MS and complementary approaches have revealed that alterations in PTMs not only serve as early indicators of neuronal dysfunction but may also represent potential therapeutic and diagnostic targets in neurodegeneration^10^ ^,^ ^11^ ^,^ ^12^. By enabling spatially resolved detection of protein isoforms and modified peptides, MALDI-MS thus contributes to a better understanding of disease-specific molecular signatures and their clinical implications^13^ ^,^ ^14^ ^,^ ^15^ ^,^ ^16^.

The MALDI technique is often performed using time-of-flight analyzers (MALDI-TOF)^14^ ^,^ ^16^, which are widely accessible and offer greater speed, lower cost, and often higher accuracy compared to earlier methods^17^. Compared to electrospray ionization mass spectrometry (ESI-MS), MALDI-MS provides advantages such as faster data acquisition, no cross-contamination, greater ion source stability, and more straightforward data interpretation^15^. Thus, the application of MALDI-MS in the analysis of AD biomarkers has proven to be highly effective.

This technique has also been instrumental in the study of Parkinson’s disease, revealing key proteomic and lipidomic changes, such as alpha-synuclein aggregation and dopamine metabolism dysfunctions^6^. Additionally, MALDI-MS has facilitated the identification of region-specific metabolic alterations in various neurological disorders, such as multiple sclerosis and glioblastoma^13^. It has also been applied in the investigation of spinal cord injuries and neuroinflammatory conditions, being used to map protein and lipid changes associated with neuronal repair and immune activation^13^. These findings reinforce the potential of MALDI-MS as a powerful tool to characterize the molecular landscape of neurological diseases and to guide the development of innovative diagnostic and therapeutic strategies.

Currently, the field of mass spectrometry lacks a comprehensive review that consolidates previous studies on the application of MALDI-MS in the proteomic analysis of neurological tissue samples in AD. This gap in the literature hinders a clear understanding of the advancements, challenges, and future directions in this area of research. The present scoping review aims to map the existing literature on the application of MALDI-MS in the proteomic analysis of brain tissues in AD. By identifying, describing, and synthesizing prior studies, this review seeks to provide an overview of the role of MALDI-MS in characterizing proteomic alterations associated with this pathology.

### Objective

To map and synthesize the existing literature on the use of MALDI-MS in the proteomic analysis of neurological tissues in AD, with a focus on its contributions to the identification of disease-related protein alterations, post-translational modifications, and biomarker discovery.

## METHODS

### Study design and protocol

A scoping review was conducted following the Joanna Briggs Institute (JBI) Manual for Evidence Synthesis^17^ and structured according to the recommendations of the Preferred Reporting Items for Systematic Reviews and Meta-Analyses extension for Scoping Reviews (PRISMA-ScR). The study protocol was prospectively registered on the Open Science Framework platform on March 25, 2025, under the DOI 10.17605/OSF.IO/WNXJG.

### Review question

What has been reported regarding the application of MALDI-MS in the proteomic analysis of neurological tissues in AD?

### Search strategy

Two authors independently conducted an exploratory search in the United States National Library of Medicine (PubMed)/ Medical Literature Analysis and Retrieval System Online (MEDLINE), Embase, and Scopus databases from inception to March 2025. The search strategy employed in this study consisted of the following terms: (“Alzheimer’s disease” OR “Alzheimer disease” OR “AD” OR “Dementia of the Alzheimer type” OR “DAT” OR “Senile Dementia of the Alzheimer Type” OR “SDAT” OR “Primary Degenerative Dementia”) AND (“Matrix-assisted laser desorption/ionization” OR MALDI OR “MALDI-TOF”) AND (“Neurological tissue” OR “Neural tissue” OR “Brain tissue” OR “Spinal cord tissue” OR “CNS tissue” OR “Peripheral nerve tissue” OR “Glial tissue” OR “Cerebral tissue” OR “Brain biopsy” OR “Spinal cord biopsy” OR “Neural biopsy” OR “rat brain” OR “mice brain” OR “Frozen brain tissue” OR “FFPE brain tissue”). The identified records were imported into Zotero (Corporation for Digital Scholarship, George Mason University). After duplicate removal, titles and abstracts were screened based on predefined eligibility criteria. Subsequently, the full texts of the remaining articles were assessed for inclusion. Additional studies were identified by reviewing the reference lists of the included articles, previous meta-analyses, and relevant reviews. Any disagreements were resolved by a third reviewer.

### Eligibility criteria

This scoping review included studies that utilized MALDI-MS for proteomic analysis in AD involving neurological tissues, including brain, spinal cord, and peripheral nervous system tissues. Eligible studies were required to have employed MALDI-MS for the identification, quantification, or analysis of post-translational modifications of proteins. Both experimental and observational studies - such as case-control, cohort, or cross-sectional designs - were included. No restrictions were applied regarding the date of publication, in order to provide a comprehensive overview of both historical and recent advances. Studies that focused exclusively on lipidomics, metabolomics, or glycomics without proteomic analysis were excluded, as were those investigating neurodegenerative diseases unrelated to AD. Research involving non-neurological tissues, such as blood or cerebrospinal fluid, was also excluded unless directly linked to the analysis of neurological tissue.

### Concept

This scoping review focuses on the application of MALDI-MS as a proteomic tool in the context of AD. Specifically, it explores how MALDI-MS has been used to identify and characterize protein alterations in neurological tissues, including beta-amyloid proteoforms and post-translational modifications of tau protein - both hallmark features of AD pathology. The review emphasizes MALDI-MS’s high spatial resolution and its ability to perform region-specific molecular profiling, which contributes to a better understanding of disease mechanisms and supports the discovery of early biomarkers.

### Context

Research settings that applied MALDI-MS for proteomic investigations of neurological tissues in AD, regardless of geographic location or publication date. The review encompasses both historical and recent studies, including observational and experimental designs, with no temporal or setting restrictions - but limited to tissues of the nervous system (excluding blood or cerebrospinal fluid unless directly linked to neural tissue).

### Quality assessment

Although formal risk of bias (ROB) assessments using tools such as ROB-1 or ROB-2 were not conducted, in accordance with the JBI Manual for Evidence Synthesis - which does not mandate such evaluations for scoping reviews - the methodological heterogeneity of the included studies was systematically considered. Variations in sample type (human versus animal), tissue processing protocols, MALDI-MS instrumentation, and data acquisition strategies were carefully noted and taken into account when interpreting the findings. This recognition of heterogeneity guided both the synthesis and the reporting of results, ensuring that observed proteomic patterns were contextualized within the specific methodological frameworks of each study, thereby enhancing the rigor and reliability of the scoping review’s conclusions.

## RESULTS

**Figure 1.**
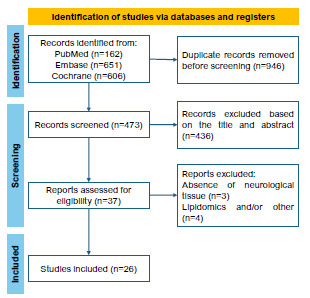
Flowchart of the study selection process.

Source: Adapted from the PRISMA 2020 flow diagram proposed by Page et al.^18^, using data from the authors’ study selection process.

The initial search retrieved 1,419 scientific records on March 25, 2024. After removing duplicates and applying eligibility criteria, 37 records were selected for full-text review (Figure 1)^8^. Of these, 26 studies were included in this scoping review. The individual characteristics of the included studies are presented in Supplementary Material Table S1.

MALDI-MS has emerged as a crucial tool for investigating AD. Its application in proteomic analysis of brain tissues has enabled high-resolution molecular characterization of pathological markers such as beta-amyloid (Aβ) plaques, tau protein modifications, lipid dysregulation, and inflammatory responses^5^ ^,^ ^13^. A summary of the main proteomic findings, organized by study type and distinguishing between human studies and animal models, is presented in Supplementary Material Table S2. Regarding Aβ, MALDI-MS has enabled detailed mapping of its proteoforms, revealing significant heterogeneity in its composition and cerebral distribution ^13^. Aβ1-40 was predominantly detected in leptomeningeal and parenchymal arterioles of the frontal and occipital cortices, while Aβ1-42 showed a more diffuse distribution in both vascular and parenchymal plaques of the hippocampus and temporal cortex, suggesting different deposition mechanisms^7^. N- and C-terminal cleavages resulted in forms such as Aβ4-42, Aβ1-39, and Aβ1-38, with substantial accumulation in affected brains, suggesting that Aβ aggregation dynamics are modulated by regional factors such as pH, enzymatic activity, and lipid microenvironment^7^. Moreover, patients with AD exhibited abnormal accumulation of citrullinated proteins^12^ and axonal loss in white matter rarefaction areas^19^, while individuals with dominantly inherited AD carrying PSEN1 V261I and A431E mutations showed cotton wool plaques predominantly composed of type I Aβ filaments^7^.

Among post-translational modifications, pyroglutamation of AβpE3-42 has received particular attention due to its increased stability and propensity for aggregation^14^ ^,^ ^20^. This modified form is more resistant to degradation and capable of forming neurotoxic oligomers, being detected in higher proportions in brains at advanced stages of AD^14^ ^,^ ^21^. The interaction between Aβ and microtubule-associated proteins (MAPs) has also been suggested as a relevant pathogenic factor^20^. MALDI-MS imaging showed colocalization between microtubule-associated protein (MAP) 2 (MAP2) and Aβ42 deposits, while MAP1A and MAP1B were associated with regions of Aβ40 accumulation. Proteomic analyses further demonstrated that Tau physiologically interacts with several MAPs, tubulins, and synaptic proteins, and that alterations in this interactome may contribute to cytoskeletal disorganization and neuronal loss observed in AD^22^ ^,^ ^23^. Since MAP2 is essential for the integrity of the neuronal cytoskeleton, its dysfunction may trigger synaptic loss and neuronal degeneration - central features of AD^1^.

Animal models such as five familial Alzheimer’s disease (5XFAD) mice support these findings^24^. Using high spatial resolution MALDI-MS, researchers were able to map the progressive accumulation of Aβ from two to six months of age and quantify modifications such as AβpE3-42, reinforcing its relevance as an early therapeutic target^24^. MALDI-MS has detected the role of photo-oxygenation in facilitating the microglial degradation of Aβ peptide in model animal mice^25^. Additionally, AD exhibited extracellular and vascular deposition of N-terminally elongated Aβ-6/-3-x peptides^21^. Although human samples directly reflect AD pathology, proteomic comparisons are confounded by heterogeneity in post-mortem intervals, disease severity, comorbidities, and patient age^26^; conversely, transgenic mouse models provide highly standardized samples with consistent pathology progression, genetic background, and tissue processing, thereby minimizing inter-individual variability and allowing proteomic changes to be more specifically attributed to amyloid pathology^27^.

Regarding tau protein, MALDI-MS identified proteomic signatures that distinguish brain regions more vulnerable to pathology^26^. Differences in tau ubiquitination and interactions with inflammatory proteins have been observed, suggesting that AD progression may depend on regional factors^27^. MALDI-MS has also enabled mapping of tau progression from initial deposits in the entorhinal cortex and hippocampus to widespread neocortical involvement in more advanced stages^28^ ^,^ ^29^. However, only a limited number of proteomic studies have sought to distinguish Alzheimer’s disease from other forms of dementia or neurodegenerative disorders^30^. This gap is critical, as overlapping clinical, pathological, and genetic features among neurodegenerative diseases hinder accurate diagnosis based on symptoms or imaging alone^26^ ^,^ ^30^.

MALDI-MS has revolutionized the study of AD by enabling high-resolution spatial mapping of proteomic, lipidomic, and metabolomic changes^28^.The integration of these approaches has provided new insights into the multifactorial nature of the disease, including the detection of hemoglobin-derived peptides associated with oxidative stress and neurotoxicity^30^ ^,^ ^31^. In the future, leveraging MALDI’s spatial resolution capabilities will be essential for biomarker discovery and the development of more precise therapeutic strategies^3^ ^,^ ^6^. These results clearly demonstrate that MALDI-MS imaging is a robust tool for routine molecular imaging of proteins and immunoprecipitation of Aβ^9^, making it a valuable asset in biomedical research, although its full capabilities are yet to be fully realized^24^ ^,^ ^32^.

## DISCUSSION

This scoping review aimed to synthesize the available evidence on the application of MALDI-MS in the proteomic analysis of neurological tissues, with a focus on the investigation of AD. From the 1,419 records initially identified, 26 studies met the eligibility criteria and were analyzed in depth, revealing how MALDI-MS has contributed to the molecular mapping of pathological changes associated with AD, including Aβ deposition, tau protein dysfunctions, alterations in lipid metabolism, inflammatory processes, and post-translational modifications^3^ ^,^ ^6^ ^,^ ^10^ ^,^ ^20^.

Analysis and treatment of AD face significant challenges due to limited blood-brain barrier permeability and inadequate drug distribution within the brain^1^. Typically, a TOF analyzer is coupled with the MALDI-MS ion source to enable measurement of intact peptides^14^, consistent with the methodologies used in the studies reviewed. One of the major outcomes identified across the reviewed studies was the capacity of MALDI-MSI to discriminate and spatially resolve distinct Aβ proteoforms and their PTMs within brain tissue^32^. Early demonstrations of this capability were provided by Stoeckli et al.^28^, who visualized amyloid beta peptides in mouse brain sections using mass spectrometry, establishing foundational methods for subsequent proteomic mapping in AD models^6^ ^,^ ^10^ ^,^ ^20^. Studies such as those by Toyama et al.^6^ and Carlred et al.^10^ demonstrated the heterogeneity of these species, with Aβ1-40 more concentrated in leptomeningeal and parenchymal arterioles of the frontal and occipital cortices, and Aβ1-42 distributed across vascular and parenchymal plaques of the hippocampus and temporal cortex, suggesting distinct deposition mechanisms. Additionally, cleaved variants such as Aβ4-42, Aβ1-39, and Aβ1-38 were detected in specific brain regions, indicating a role of the local microenvironment - including pH, enzymatic activity, lipid composition, and PTMs - in modulating the aggregation of these species^32^.

The presence of the pyroglutamylated form AβpE3-42, highlighted by Kaya et al.,^32^ is particularly relevant due to its neurotoxic potential, increased resistance to degradation, and high capacity to form stable oligomers. PTMs such as phosphorylation, ubiquitination, and citrullination also modulate tau and Aβ pathology and may contribute to synaptic dysfunction, neurodegeneration, and region-specific vulnerability^6^ ^,^ ^7^ ^,^ ^32^. Findings from animal models, such as 5XFAD mice, support this relevance by demonstrating early and progressive accumulation of AβpE3-42 with aging^7^ ^,^ ^32^, reinforcing the need for intervention strategies during the initial stages of the disease. However, although human samples directly reflect AD’s pathology, it is important to acknowledge limitations such as postmortem changes, histological processing artifacts, technical limitations of proteomic analyses, and patient heterogeneity^6^ ^,^ ^20^. Conversely, transgenic mouse models provide highly standardized samples with consistent pathology progression, genetic background, and tissue processing, minimizing inter-individual variability, but they do not fully recapitulate the sporadic forms of AD that account for more than 90% of cases^6^ ^,^ ^10^ ^,^ ^32^.

Another key point was the interaction between Aβ and MAPs, especially MAP2, whose co-localization with Aβ42 deposits was demonstrated by Toyama et al.^6^. MAP2, being essential for neuronal cytoskeleton stability, may contribute to synaptic loss and neurodegeneration when affected by such pathological interactions, as suggested by Michno et al.^7^ and Uras et al.^24^. Meanwhile, MAP1A and MAP1B were found to be associated with Aβ40, indicating that different Aβ isoforms may interact specifically with distinct neuronal structural components^6^ ^,^ ^7^.

This study has notable limitations. First, the use of post-mortem human brain samples is inherently constrained by potential alterations due to post-mortem interval, histological processing, and technical limitations, which may affect the detection of subtle proteomic and post-translational modifications^6^ ^,^ ^20^. Second, while transgenic mouse models provide standardized and reproducible samples, they predominantly reflect familial forms of AD and may not fully capture the pathophysiological complexity of sporadic AD, which accounts for over 90% of cases^10^ ^,^ ^32^. Third, methodological heterogeneity across included studies, including differences in MALDI-MS instrumentation, sample preparation, and spatial resolution, limits direct comparisons and generalizability of the findings^10^ ^,^ ^28^ ^,^ ^32^. Finally, the absence of formal ROB assessment, typical of scoping reviews, may influence the interpretation of the reported proteomic patterns^17^. These limitations highlight the need for careful consideration when translating MALDI-MS findings from preclinical models and post-mortem tissues into clinical applications.

This scoping review highlights the essential role of MALDI-MS in the proteomic analysis of neurological tissues affected by AD. The included studies demonstrated that MALDI-MS enables detailed spatial characterization of biomolecules associated with AD pathogenesis, such as different isoforms of beta-amyloid peptides and modified forms of tau protein^10^ ^,^ ^32^ ^,^ ^33^. Proteomics, as a disease-driven approach, has proven valuable in elucidating molecular mechanisms underlying neurodegeneration^6^ ^,^ ^7^. For instance, preclinical studies in mouse models have characterized alterations in brain protein expression using advanced proteomic techniques^33^, while disease-driven human studies provided insights into pathological protein networks and post-translational modifications^34^. The technique not only provides insights into the molecular complexity of the disease, but also contributes to the identification of early therapeutic targets and potential diagnostic biomarkers^33^ ^,^ ^35^ ^,^ ^36^. From a clinical perspective, the advancement of MALDI-MS holds significant implications for the early detection, monitoring, and individualized treatment of AD^20^ ^,^ ^37^. As this technology becomes more refined and accessible, it is poised to bridge the gap between molecular neuropathology and patient-centered care^33^. In particular, MALDI-MS could support the development of minimally invasive diagnostic tools, guide biomarker-driven clinical trials, and inform therapeutic strategies tailored to the molecular profile of individual patients^35^ ^,^ ^37^.

Future MALDI-MS studies should prioritize the standardization of tissue collection, processing, and storage to minimize technical variability and enhance reproducibility across laboratories^6^ ^,^ ^10^ ^,^ ^33^. Optimizing pre-analytical factors, such as post-mortem interval, fixation methods, and sectioning protocols, is essential to ensure consistent detection of subtle proteomic changes and PTMs^6^ ^,^ ^32^ ^,^ ^33^ ^,^ ^34^. Improvements in the spatial resolution of MALDI-MS imaging, especially when combined with multi-modal approaches like co-registration with immunohistochemistry or imaging mass cytometry, will enable precise mapping of protein isoforms and PTMs across vulnerable brain regions^6^ ^,^ ^7^ ^,^ ^32^ ^,^ ^33^ ^,^ ^34^. Integrating these techniques enhances the identification of region-specific molecular signatures that drive disease progression, fostering a more nuanced understanding of neurodegenerative mechanisms in both sporadic and familial forms of AD^6^ ^,^ ^7^ ^,^ ^10^ ^,^ ^32^ ^,^ ^36^. Minimally invasive detection strategies, including the analysis of exosomes, cerebrospinal fluid, or blood-derived components, could bridge the gap between tissue-based discoveries and patient-centered applications^6^ ^,^ ^33^. Furthermore, insights from MALDI-MS-guided proteomic mapping may inform the design of advanced therapeutic systems, such as targeted nano-missiles capable of crossing the blood-brain barrier to reduce neuroinflammation and provide neuroprotection, as demonstrated by He et al.^37^. Additionally, comparative studies between sporadic and familial AD are important to elucidate disease heterogeneity, revealing distinct proteomic and PTM profiles that underlie differential vulnerability patterns and progression trajectories^6^ ^,^ ^7^ ^,^ ^10^. Collectively, these strategies will advance mechanistic understanding, support biomarker-driven clinical trials, and guide the development of personalized therapeutic approaches^6^ ^,^ ^19^ ^,^ ^33^. By enabling early detection, monitoring, and individualized treatment, MALDI-MS has the potential to bridge molecular neuropathology and patient-centered care, ultimately fostering more precise, predictive, and personalized strategies for the diagnosis and treatment of AD^35^ ^,^ ^37^.

## Supplementary Material

Supplementary information are available at https://www.demneuropsy.org/wp-content/uploads/2026/05/DN-2025.0437-Supplementary-Material.docx

Supplementary DOCX

## Data Availability

The datasets generated and/or analyzed during the current study are available from the corresponding author upon reasonable request.
