# Supplementary material for: Matrix-assisted laser desorption/ionization mass spectrometry (MALDI) in Alzheimer’s disease: a scoping review of proteomic alterations in neurological tissues
Source: Dement Neuropsychol. 2026 Jul 24;20:e20250437. doi: 10.1590/1980-5764-DN-2025-0437 (PMC13403519; doi:10.1590/1980-5764-DN-2025-0437)
Supplement: Supplementary DOCX [file 1980-5764-dn-20-e20250437-sppl.docx]

**Supplementary Material**

**Table S1.** Baseline characteristics of included studies

| Study | Country | Type of Study | Neurological Tissue | Type of Sample | Analyser | Matrix |
| --- | --- | --- | --- | --- | --- | --- |
|  |  |  |  |  |  |  |
| Carlred  2016 | Sweden | Experimental | Brain | Animal | TOF* | CHCA^+^ |
| Castaño  2013 | Argentina | Experimental | Brain | Human | TOF* | — |
| Esler  2000 | U.S.A | Experimental | Brain | Human | TOF* | — |
| Gobom  2024 | Sweden | Review | Brain | Human | TOF* | — |
| He  2022 | China | Experimental | Brain | Animal | TOF* | — |
| Hoq  2024 | U.S.A | Experimental | Brain | Human | TOF* | — |
| Ishigami  2005 | Japan | Experimental | Brain | Human | TOF* | CHCA^+^ |
| Kaya  2017 | Sweden | Experimental | Brain | Animal | TOF* | 1,5-DAN^§^ |
| Kaya  2018 | Sweden | Experimental | Brain | Animal | TOF* | 1,5-DAN^§^ |
| Michno  2019 | Sweden | Experimental | Brain | Human and Animal | TOF* | 2,5-DHA^\|^**^\|^** |
| Murray  2018 | United Kingdom | Experimental | Brain | Human | TOF* | CHCA^+^ |
| Noor  2022 | Germany | Experimental | Brain | Human | TOF* | SA^¶^ |
| Ozawa  2021 | Japan | Experimental | Brain | Human and Animal | TOF* | CHCA^+^ |
| Poljak  2004 | Australia | Experimental | Brain | Human | TOF* | CHCA ^+^ |
| Portelius  2009 | Sweden | Experimental | Brain | Animal | TOF* | CHCA^+^ |
| Richard  2019 | Germany | Experimental | Brain | Animal | TOF* | SA^¶^ |
| Riederer  2008 | Sweden | Experimental | Brain | Human | TOF* | CHCA^+^ |
| Schubert  2016 | Australia | Review | Brain | Human and Animal | TOF* | — |
| Schulenborg  2006 | Germany | Review | Brain | Human and Animal | TOF* | — |
| Sinsky  2020 | Slovakia | Experimental | Brain | Human and Animal | TOF* | CHCA^+^ |
| Sizova  2007 | France | Experimental | Brain | Animal | TOF* | CHCA^+^ |
| Stoeckli  2002 | Switzerland | Experimental | Brain | Animal | TOF* | SA^¶^ |
| Tabaton  2006 | Italy | Experimental | Brain | Human | TOF * | — |
| Toyama  2024 | Japan | Experimental | Brain | Human | TOF* | DHB^**^ |
| Uras  2023 | Turkiye | Experimental | Brain | Animal | TOF* | DHB^**^ |
| Wirths  2024 | Germany | Experimental | Brain | Human and Animal | TOF* | CHCA^+^ |
| Zellner  2009 | Germany | Review | Brain | Human and Animal | — | — |

Notes: *Time-of-Flight; +Alpha-Cyano-4-Hydroxycinnamic Acid; § 1,5-diaminonaphthalene; **||:** 2,5-Dihydroxyacetophenone; ¶Sinapinic acid; **2,5-dihydroxybenzoic acid.

**Table S2**. This table summarizes the main proteomic findings in Alzheimer’s disease from human and animal studies, including amyloid-beta and tau heterogeneity, post-translational modifications, and alterations in synaptic, mitochondrial, and cytoskeletal proteins.

| **Study (Year)** | **Technique** | **Main Proteomic Findings** | **Pathological / Clinical Implications** |
| --- | --- | --- | --- |
| **Human Studies** |  |  |  |
| Toyama et al., 2024 (6) | Integrated spatial multi-omics combining matrix-assisted laser desorption/ionization mass spectrometry imaging and transcriptomics | Regional heterogeneity of amyloid-beta, tau, and lipid species; glial activation signatures co-localized with amyloid plaques | Region-specific metabolic and inflammatory proteomic networks in Alzheimer’s disease cortex |
| Ikegawa et al., 2023 (3) | Matrix-assisted laser desorption/ionization mass spectrometry imaging | Spatially resolved profiles of amyloid-beta 1–42, pyroglutamate-modified amyloid-beta 3–42, tau fragments, and oxidized peptides | Enables molecular mapping of plaque polymorphism and protein oxidation |
| Noor et al., 2022 (20) | Liquid chromatography–tandem mass spectrometry proteoform profiling | Rapidly progressive cases show enrichment of truncated and pyroglutamated amyloid-beta | Distinct amyloid-beta proteoforms underlie accelerated neurodegeneration |
| Wirths et al., 2024 (21) | Immunoblotting combined with mass spectrometry | A disintegrin and metalloproteinase with thrombospondin motifs 4 (ADAMTS4) generates N-terminally elongated amyloid-beta species | Indicates alternative amyloidogenic processing beyond β/γ-secretase pathways |
| Ishigami et al., 2005 (12) | Liquid chromatography–mass spectrometry and enzymatic assays | Accumulation of citrullinated proteins generated by peptidylarginine deiminase | Post-translational deimination contributes to protein misfolding and aggregation |
| Poljak et al., 2004 (31) | Liquid chromatography–tandem mass spectrometry quantification | Elevated hemorphins (hemoglobin-derived peptides) | Reflects vascular dysfunction and oxidative stress in Alzheimer’s disease brain |
| Castaño et al., 2013 (22) | Liquid chromatography–tandem mass spectrometry proteomics | Decrease of myelin and metabolic proteins; increase of oxidative markers | White matter degeneration involves distinct proteomic remodeling |
| Sinsky et al., 2020 (23) | Affinity-based proteomics and interactome analysis | Defined physiological tau interactome, including cytoskeletal and synaptic proteins | Loss of normal tau interactions contributes to tauopathy progression |
| Hoq et al., 2024 (19) | Cryogenic electron microscopy–based structural proteomics | Distinct amyloid-beta 42 conformers in “cotton-wool” plaques and specific tau filament morphologies | Structure-dependent proteomic heterogeneity in inherited Alzheimer’s disease |
| Gobom et al., 2024 (9) | Targeted liquid chromatography–tandem mass spectrometry using multiple reaction monitoring | Quantified amyloid-beta isoforms, tau fragments, and neurogranin | Provides standardized, quantitative proteomic biomarkers for Alzheimer’s disease diagnosis |
| **Animal Model Studies** |  |  |  |
| Carlred et al., 2016 (10) | Matrix-assisted laser desorption/ionization imaging mass spectrometry | Spatial heterogeneity of amyloid-beta 1–42 and pyroglutamate-modified amyloid-beta 3–42 within plaques | Analogous amyloid-beta polymorphism to human Alzheimer’s disease pathology |
| Kaya et al., 2017 (13) | Matrix-assisted laser desorption/ionization mass spectrometry imaging | Altered sphingolipids near amyloid-beta plaques | Lipid dysregulation contributes to neurotoxicity and plaque growth |
| Kaya et al., 2018 (32) | Multimodal matrix-assisted laser desorption/ionization mass spectrometry imaging combined with fluorescence microscopy | Co-localization of amyloid-beta proteoforms with oxidized lipids and lipofuscin | Links lipid peroxidation and oxidative stress to plaque maturation |
| Uras et al., 2023 (24) | Matrix-assisted laser desorption/ionization mass spectrometry imaging | Early proteomic alterations in synaptic, mitochondrial, and cytoskeletal proteins | Proteomic remodeling precedes visible amyloid pathology |
| Stoeckli et al., 2002 (28) | Matrix-assisted laser desorption/ionization imaging | First in situ visualization of amyloid-beta spatial distribution | Established feasibility of direct proteomic imaging in Alzheimer’s disease models |
| Portelius et al., 2009 (11) | Liquid chromatography–tandem mass spectrometry | Gamma-secretase inhibition shifts amyloid-beta isoform pattern toward shorter peptides | Supports isoform-specific targeting for therapeutic modulation |
| Ozawa et al., 2021 (25) | Photo-oxygenation combined with liquid chromatography–tandem mass spectrometry | Reduced amyloid-beta aggregation and restored synaptic proteins after catalytic photo-therapy | Demonstrates proteomic reversal of amyloidosis |
| Sizova et al., 2007 (27) | Two-dimensional difference gel electrophoresis proteomics | Altered metabolic, chaperone, and cytoskeletal proteins | Early oxidative and folding stress drive pathology |
| Murray et al., 2018 (26) | Regional liquid chromatography–tandem mass spectrometry proteomics | Preserved proteome in presubiculum region | Identifies neuroprotective regional protein signatures |
| He et al., 2022 (37) | Quantitative proteomics | Restoration of mitochondrial and antioxidant proteins | Confirms anti-inflammatory and neuroprotective proteomic effects |
| Schulenborg et al., 2006 (29) | Various proteomic platforms | Overview of disease-driven proteomics in neurodegeneration | Defines transition to functional and spatial proteomics |
| Zellner et al., 2009 (30) | Proteomics in dementia research | Emphasized post-translational modifications and translational reproducibility | Highlights challenges for biomarker standardization |
| Schubert et al., 2016 (33) | Matrix-assisted laser desorption/ionization mass spectrometry imaging applications | Integration of imaging and neuroproteomics approaches | Established mass spectrometry imaging as key in neurodegenerative proteomics |
| Riederer et al., 2008 (34) | Infrared maleimide dye labeling | Detection of cysteine oxidation and modification | Improved identification of redox-sensitive proteoforms |
| Tabaton and Gambetti, 2006 (35) | Soluble amyloid-beta species | Focused on toxic soluble amyloid-beta oligomers | Predicted proteomic relevance of soluble amyloid-beta in synaptic failure |
| Esler et al., 2000 (36) | In vitro peptide biochemistry | Described “dock-lock” amyloid-beta propagation mechanism | Provided mechanistic basis for later proteomic and structural studies |
